# Retraction Note: The effects of sildenafil citrate on intrauterine growth restriction: a systematic review and meta-analysis: Wassim Y. Almawi

**DOI:** 10.1186/s12884-025-08395-1

**Published:** 2025-10-27

**Authors:** Yenlik Rakhanova, Wassim Y. Almawi, Gulzhanat Aimagambetova, Dieter Riethmacher

**Affiliations:** 1 School of Medicine, Nazarbayev University, Astana, 010000 Kazakhstan; 2 Faculté Des Sciences de Tunis, Université de Tunis - El Manar, Tunis, Tunisia; 3 Department of Biomedical Sciences, School of Medicine, Nazarbayev University, Astana, 010000 Kazakhstan; 4 Department of Surgery, School of Medicine, Nazarbayev University, Astana, 010000 Kazakhstan

**Retraction Note: BMC Pregnancy and Childbirth 23, 409 (2023)**

**https://doi.org/10.1186/s12884-023-05747-7**

This article has been retracted by the Editor. Following publication, concerns were raised that some of the included studies have subsequently been retracted or have published Expressions of Concern. Although a reanalysis by the authors found that the removal of these included studies from their systematic review and meta-analysis, does not alter the conclusions of the article, an investigation by the Publisher identified additional methodological concerns regarding the study. These concerns are that the conclusion that Sildenafil citrate prolongs pregnancy in IUGR cases does not appear to be supported by the data, as the 95% CI reported in Fig. 3, Panel B crosses 0, suggesting that the results are not statistically significant. In addition, post publication peer review identified methodological concerns regarding the comparison of absolute birthweights without adjustment for neonatal age. In light of these concerns, the Editor no longer has confidence in the findings and conclusions of this systematic review and meta-analysis.

Author Gulzhanat Aimagambetova disagrees with this retraction. Authors Yenlik Rakhanova, Wassim Y. Almawi and Dieter Riethmacher did not respond to correspondence from the Publisher about this retraction.

